# 2-(4-Fluoro­phen­yl)-3-methyl­sulfanyl-5-phenyl-1-benzofuran

**DOI:** 10.1107/S1600536809041713

**Published:** 2009-10-17

**Authors:** Hong Dae Choi, Pil Ja Seo, Byeng Wha Son, Uk Lee

**Affiliations:** a Department of Chemistry, Dongeui University, San 24 Kaya-dong Busanjin-gu, Busan 614-714, Republic of Korea; b Department of Chemistry, Pukyong National University, 599-1 Daeyeon 3-dong, Nam-gu, Busan 608-737, Republic of Korea

## Abstract

In the title compound, C_21_H_15_FOS, the 4-fluoro­phenyl ring is rotated out of the benzofuran plane, making a dihedral angle of 24.3 (1)°. The dihedral angle between the phenyl ring and the benzofuran plane is 28.3 (1)°. The crystal structure may be stabilized by two very weak aromatic π–π inter­actions between the furan and the benzene rings of neighbouring benzofuran systems; the centroid–centroid distances are 3.909 (4) and 4.028 (4) Å.

## Related literature

For the crystal structures of similar 2,5-diaryl-3-methyl­sulfanyl-1-benzofuran derivatives, see: Choi, Seo *et al.* (2006 ▶); Choi, Woo *et al.* (2006 ▶). For natural products with benzofuran ring systems, see: Akgul & Anil (2003 ▶); Soekamto *et al.* (2003 ▶); von Reuss & König (2004 ▶).
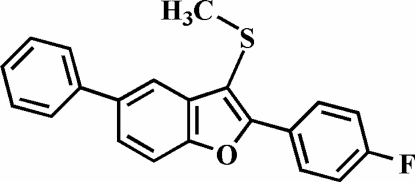


## Experimental

### 

#### Crystal data

C_21_H_15_FOS
*M* *_r_* = 334.39
Monoclinic, 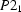

*a* = 10.621 (6) Å
*b* = 7.192 (4) Å
*c* = 11.642 (6) Å
β = 116.076 (5)°
*V* = 798.7 (8) Å^3^
*Z* = 2
Mo *K*α radiation
μ = 0.22 mm^−1^
*T* = 173 K
0.50 × 0.42 × 0.33 mm

#### Data collection

Bruker SMART APEXII CCD diffractometer
Absorption correction: multi-scan (*SADABS*; Bruker, 2009 ▶) *T*_min_ = 0.893, *T*_max_ = 0.932
7602 measured reflections
3339 independent reflections
3147 reflections with *I* > 2σ(*I*)
*R*_int_ = 0.095

#### Refinement

*R*[*F* ^2^ > 2σ(*F* ^2^)] = 0.050
*wR*(*F* ^2^) = 0.133
*S* = 1.04
3339 reflections
218 parameters
1 restraint
H-atom parameters constrained
Δρ_max_ = 0.41 e Å^−3^
Δρ_min_ = −0.44 e Å^−3^
Absolute structure: Flack (1983), 1362 Friedel pairs
Flack parameter: 0.00 (9)

### 

Data collection: *APEX2* (Bruker, 2009 ▶); cell refinement: *SAINT* (Bruker, 2009 ▶); data reduction: *SAINT*; program(s) used to solve structure: *SHELXS97* (Sheldrick, 2008 ▶); program(s) used to refine structure: *SHELXL97* (Sheldrick, 2008 ▶); molecular graphics: *ORTEP-3* (Farrugia, 1997 ▶) and *DIAMOND* (Brandenburg, 1998 ▶); software used to prepare material for publication: *SHELXL97*.

## Supplementary Material

Crystal structure: contains datablocks I. DOI: 10.1107/S1600536809041713/vm2006sup1.cif

Structure factors: contains datablocks I. DOI: 10.1107/S1600536809041713/vm2006Isup2.hkl

Additional supplementary materials:  crystallographic information; 3D view; checkCIF report

## Figures and Tables

**Fig. 1.**
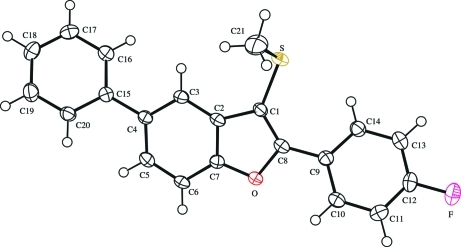
The molecular structure of the title compound with the atom numbering scheme. Displacement ellipsoids are drawn at the 50% probability level. H atoms are presented as a small spheres of arbitrary radius.

**Fig. 2.**
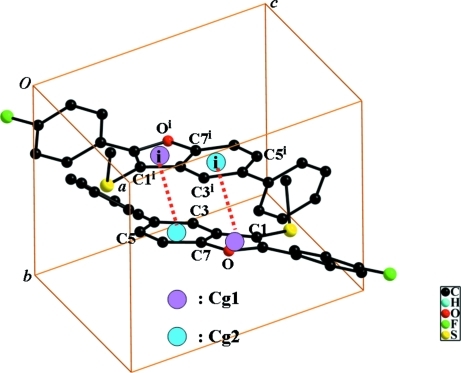
π···π interactions (dotted lines) in the crystal structure of the title compound. Cg denotes the ring centroids. [Symmetry codes: (i) - x + 1, y - 1/2, - z + 1.]

## supplementary crystallographic information

### Comment

Molecules containing the benzofuran skeleton constitute a major group of naturally-occurring compounds that are of a remarkable interest because of their biological activities (Akgul & Anil, 2003; Soekamto *et al.*, 2003; von Reuss & König, 2004). As a part of our continuing studies of the effect of side chain substituents on the solid state structures of 2,5-diaryl-3-methylsulfanyl-1-benzofuran analogues (Choi, Seo *et al.*, 2006; Choi, Woo *et al.*, 2006), we report the crystal structure of the title compound (Fig. 1).

The benzofuran unit is essentially planar, with a mean deviation of 0.011 (2) Å from the least-squares plane defined by the nine constituent atoms. The 4-fluorophenyl ring is rotated out of the benzofuran plane, with a dihedral angle of 24.3 (1)°. The dihedral angle between the phenyl ring and the benzofuran plane is 28.3 (1)°. The molecular packing (Fig. 2) is stabilized by two aromatic π···π interactions between the furan and the benzene rings of adjacent benzofuran molecules, with a Cg1···Cg2^i^ and a Cg2···Cg1^i^ distances of 3.909 (4) and 4.028 (4) Å, respectively (Cg1 and Cg2 are the centroids of the C1/C2/C7/O/C8 furan ring and the C2–C7 benzene ring, respectively, symmetry code i: - *x* + 1, *y* - 1/2, - *z* + 1).

### Experimental

Zinc chloride (273 mg, 2.0 mmol) was added to a stirred solution of 4-phenylphenol (340 mg, 2.0 mmol) and 2-chloro-4'-fluoro-2-methylsulfanylacetophenone (437 mg, 2.0 mmol) in dichloromethane (30 ml) at room temperature, and stirring was continued at the same temperature for 40 min. The reaction was quenched by the addition of water and the organic layer separated, dried over magnesium sulfate, filtered and concentrated in vacuum. The residue was purified by column chromatography (carbon tetrachloride) to afford the title compound as a colorless solid [yield 68%, m.p. 431–432 K; *R*_f_ = 0.71 (carbon tetrachloride)]. Single crystals suitable for X-ray diffraction were prepared by evaporation of a solution of the title compound in tetrahydrofuran at room temperature.

### Refinement

All H atoms were positioned geometrically and refined using a riding model, with C—H = 0.93 Å for the aryl H atoms and 0.96 Å for the methyl H atoms, and with U_iso_(H) = 1.2U_eq_(C) for the aryl H atoms and 1.5U_eq_(C) for the methyl H atoms.

### Crystal data

**Table:**

C_21_H_15_FOS	*F*(000) = 348
*M**_r_* = 334.39	*D*_x_ = 1.390 Mg m^−^^3^
Monoclinic, *P*2_1_	Mo *K*α radiation, λ = 0.71073 Å
Hall symbol: P 2yb	Cell parameters from 5096 reflections
*a* = 10.621 (6) Å	θ = 3.4–27.5°
*b* = 7.192 (4) Å	µ = 0.22 mm^−^^1^
*c* = 11.642 (6) Å	*T* = 173 K
β = 116.076 (5)°	Block, colorless
*V* = 798.7 (8) Å^3^	0.50 × 0.42 × 0.33 mm
*Z* = 2	

### Data collection

**Table:**

Bruker SMART APEXII CCD diffractometer	3339 independent reflections
Radiation source: Rotating Anode	3147 reflections with *I* > 2σ(*I*)
HELIOS	*R*_int_ = 0.095
Detector resolution: 10.0 pixels mm^-1^	θ_max_ = 27.5°, θ_min_ = 2.0°
φ and ω scans	*h* = −12→13
Absorption correction: multi-scan (*SADABS*; Bruker, 2009)	*k* = −9→9
*T*_min_ = 0.893, *T*_max_ = 0.932	*l* = −14→15
7602 measured reflections	

### Refinement

**Table:**

Refinement on *F*^2^	Secondary atom site location: difference Fourier map
Least-squares matrix: full	Hydrogen site location: difference Fourier map
*R*[*F*^2^ > 2σ(*F*^2^)] = 0.050	H-atom parameters constrained
*wR*(*F*^2^) = 0.133	*w* = 1/[σ^2^(*F*_o_^2^) + (0.0945*P*)^2^] where *P* = (*F*_o_^2^ + 2*F*_c_^2^)/3
*S* = 1.04	(Δ/σ)_max_ = 0.001
3339 reflections	Δρ_max_ = 0.41 e Å^−^^3^
218 parameters	Δρ_min_ = −0.43 e Å^−^^3^
1 restraint	Absolute structure: Flack (1983), 1362 Friedel pairs
Primary atom site location: structure-invariant direct methods	Flack parameter: 0.00 (9)

### Special details

**Table:**

Geometry. All esds (except the esd in the dihedral angle between two l.s. planes) are estimated using the full covariance matrix. The cell esds are taken into account individually in the estimation of esds in distances, angles and torsion angles; correlations between esds in cell parameters are only used when they are defined by crystal symmetry. An approximate (isotropic) treatment of cell esds is used for estimating esds involving l.s. planes.
Refinement. Refinement of F^2^ against ALL reflections. The weighted R-factor wR and goodness of fit S are based on F^2^, conventional R-factors R are based on F, with F set to zero for negative F^2^. The threshold expression of F^2^ > 2sigma(F^2^) is used only for calculating R-factors(gt) etc. and is not relevant to the choice of reflections for refinement. R-factors based on F^2^ are statistically about twice as large as those based on F, and R- factors based on ALL data will be even larger.

### Fractional atomic coordinates and isotropic or equivalent isotropic displacement parameters (Å^2^)

**Table:**

	*x*	*y*	*z*	*U*_iso_*/*U*_eq_	
S	0.55481 (6)	0.85482 (12)	0.86991 (5)	0.03285 (19)	
F	1.25269 (13)	0.7897 (3)	1.00995 (14)	0.0411 (4)	
O	0.63814 (15)	0.7920 (2)	0.57182 (13)	0.0245 (4)	
C1	0.5612 (2)	0.8243 (3)	0.72327 (19)	0.0223 (5)	
C2	0.4412 (2)	0.8143 (3)	0.60022 (19)	0.0227 (5)	
C3	0.2962 (2)	0.8170 (3)	0.55929 (18)	0.0221 (5)	
H3	0.2591	0.8309	0.6178	0.027*	
C4	0.2085 (2)	0.7984 (3)	0.42913 (19)	0.0212 (4)	
C5	0.2683 (2)	0.7823 (4)	0.3427 (2)	0.0260 (5)	
H5	0.2089	0.7725	0.2558	0.031*	
C6	0.4106 (2)	0.7804 (4)	0.38153 (19)	0.0251 (5)	
H6	0.4485	0.7701	0.3234	0.030*	
C7	0.4948 (2)	0.7947 (3)	0.5121 (2)	0.0230 (5)	
C8	0.6759 (2)	0.8099 (3)	0.70145 (19)	0.0234 (5)	
C9	0.8275 (2)	0.8059 (3)	0.78298 (19)	0.0229 (5)	
C10	0.9163 (2)	0.7210 (4)	0.7396 (2)	0.0251 (5)	
H10	0.8786	0.6685	0.6584	0.030*	
C11	1.0589 (2)	0.7133 (4)	0.8147 (2)	0.0297 (5)	
H11	1.1177	0.6548	0.7858	0.036*	
C12	1.1119 (2)	0.7952 (4)	0.9344 (2)	0.0283 (5)	
C13	1.0284 (2)	0.8825 (4)	0.9805 (2)	0.0290 (5)	
H13	1.0674	0.9376	1.0610	0.035*	
C14	0.8855 (2)	0.8868 (4)	0.9050 (2)	0.0262 (5)	
H14	0.8273	0.9437	0.9352	0.031*	
C15	0.05328 (19)	0.7926 (3)	0.38285 (19)	0.0219 (4)	
C16	−0.0110 (2)	0.8798 (4)	0.4505 (2)	0.0260 (5)	
H16	0.0430	0.9478	0.5238	0.031*	
C17	−0.1552 (2)	0.8657 (4)	0.4092 (2)	0.0301 (5)	
H17	−0.1969	0.9239	0.4551	0.036*	
C18	−0.2363 (2)	0.7662 (4)	0.3008 (2)	0.0332 (6)	
H18	−0.3322	0.7546	0.2748	0.040*	
C19	−0.1752 (2)	0.6834 (4)	0.2304 (2)	0.0335 (6)	
H19	−0.2304	0.6199	0.1554	0.040*	
C20	−0.0313 (2)	0.6951 (4)	0.2718 (2)	0.0263 (5)	
H20	0.0094	0.6373	0.2249	0.032*	
C21	0.4797 (3)	0.6375 (5)	0.8842 (3)	0.0451 (7)	
H21A	0.5406	0.5375	0.8863	0.068*	
H21B	0.3899	0.6218	0.8123	0.068*	
H21C	0.4686	0.6371	0.9617	0.068*	

### Atomic displacement parameters (Å^2^)

**Table:**

	*U*^11^	*U*^22^	*U*^33^	*U*^12^	*U*^13^	*U*^23^
S	0.0337 (3)	0.0481 (4)	0.0206 (3)	−0.0045 (3)	0.0155 (2)	−0.0065 (3)
F	0.0241 (7)	0.0489 (10)	0.0401 (8)	0.0020 (6)	0.0050 (6)	0.0013 (8)
O	0.0244 (7)	0.0326 (9)	0.0174 (7)	0.0000 (7)	0.0101 (6)	−0.0011 (7)
C1	0.0248 (9)	0.0243 (12)	0.0181 (9)	−0.0034 (8)	0.0097 (8)	−0.0029 (9)
C2	0.0292 (10)	0.0231 (13)	0.0178 (9)	−0.0019 (9)	0.0123 (8)	−0.0015 (9)
C3	0.0262 (10)	0.0230 (12)	0.0199 (9)	−0.0009 (9)	0.0127 (8)	−0.0004 (9)
C4	0.0249 (10)	0.0196 (11)	0.0198 (9)	0.0002 (8)	0.0105 (8)	0.0002 (9)
C5	0.0297 (11)	0.0273 (12)	0.0188 (9)	0.0010 (10)	0.0087 (8)	−0.0011 (9)
C6	0.0304 (11)	0.0310 (13)	0.0169 (9)	0.0000 (10)	0.0130 (9)	−0.0013 (9)
C7	0.0238 (9)	0.0257 (12)	0.0207 (10)	−0.0008 (9)	0.0110 (8)	−0.0001 (9)
C8	0.0299 (10)	0.0227 (12)	0.0184 (9)	−0.0013 (9)	0.0113 (8)	−0.0011 (9)
C9	0.0247 (10)	0.0226 (12)	0.0222 (10)	−0.0018 (9)	0.0112 (8)	0.0012 (9)
C10	0.0305 (11)	0.0223 (11)	0.0235 (10)	−0.0015 (9)	0.0129 (9)	−0.0022 (9)
C11	0.0285 (11)	0.0291 (13)	0.0359 (12)	0.0019 (9)	0.0181 (10)	−0.0006 (11)
C12	0.0218 (10)	0.0278 (12)	0.0288 (11)	−0.0005 (9)	0.0052 (9)	0.0057 (10)
C13	0.0320 (11)	0.0289 (14)	0.0212 (10)	−0.0020 (10)	0.0071 (9)	−0.0006 (10)
C14	0.0277 (10)	0.0261 (13)	0.0242 (10)	0.0002 (10)	0.0108 (8)	−0.0017 (10)
C15	0.0235 (10)	0.0198 (11)	0.0210 (9)	0.0013 (9)	0.0086 (8)	0.0031 (9)
C16	0.0293 (10)	0.0236 (12)	0.0247 (10)	0.0017 (10)	0.0115 (8)	0.0002 (10)
C17	0.0322 (11)	0.0289 (12)	0.0361 (12)	0.0054 (11)	0.0212 (9)	0.0021 (12)
C18	0.0218 (10)	0.0351 (14)	0.0408 (13)	0.0023 (9)	0.0121 (10)	0.0021 (12)
C19	0.0276 (11)	0.0355 (14)	0.0303 (12)	−0.0006 (10)	0.0062 (9)	−0.0035 (11)
C20	0.0283 (11)	0.0272 (12)	0.0240 (10)	0.0016 (9)	0.0119 (9)	−0.0038 (10)
C21	0.0473 (15)	0.056 (2)	0.0393 (15)	−0.0026 (14)	0.0255 (13)	0.0137 (14)

### Geometric parameters (Å, °)

**Table:**

S—C1	1.752 (2)	C10—H10	0.9300
S—C21	1.795 (3)	C11—C12	1.384 (3)
F—C12	1.362 (2)	C11—H11	0.9300
O—C7	1.368 (3)	C12—C13	1.374 (3)
O—C8	1.387 (3)	C13—C14	1.380 (3)
C1—C8	1.353 (3)	C13—H13	0.9300
C1—C2	1.441 (3)	C14—H14	0.9300
C2—C7	1.381 (3)	C15—C20	1.396 (3)
C2—C3	1.398 (3)	C15—C16	1.397 (3)
C3—C4	1.393 (3)	C16—C17	1.393 (3)
C3—H3	0.9300	C16—H16	0.9300
C4—C5	1.411 (3)	C17—C18	1.375 (4)
C4—C15	1.493 (3)	C17—H17	0.9300
C5—C6	1.374 (3)	C18—C19	1.384 (4)
C5—H5	0.9300	C18—H18	0.9300
C6—C7	1.388 (3)	C19—C20	1.389 (3)
C6—H6	0.9300	C19—H19	0.9300
C8—C9	1.467 (3)	C20—H20	0.9300
C9—C10	1.391 (3)	C21—H21A	0.9600
C9—C14	1.403 (3)	C21—H21B	0.9600
C10—C11	1.378 (3)	C21—H21C	0.9600
			
C1—S—C21	100.97 (13)	C12—C11—H11	121.0
C7—O—C8	106.04 (16)	F—C12—C13	118.3 (2)
C8—C1—C2	106.57 (18)	F—C12—C11	118.9 (2)
C8—C1—S	128.05 (16)	C13—C12—C11	122.7 (2)
C2—C1—S	125.37 (15)	C12—C13—C14	118.6 (2)
C7—C2—C3	119.98 (19)	C12—C13—H13	120.7
C7—C2—C1	105.69 (18)	C14—C13—H13	120.7
C3—C2—C1	134.31 (19)	C13—C14—C9	120.4 (2)
C4—C3—C2	118.64 (19)	C13—C14—H14	119.8
C4—C3—H3	120.7	C9—C14—H14	119.8
C2—C3—H3	120.7	C20—C15—C16	118.23 (19)
C3—C4—C5	119.2 (2)	C20—C15—C4	120.47 (19)
C3—C4—C15	119.99 (19)	C16—C15—C4	121.28 (19)
C5—C4—C15	120.80 (19)	C17—C16—C15	120.5 (2)
C6—C5—C4	122.8 (2)	C17—C16—H16	119.7
C6—C5—H5	118.6	C15—C16—H16	119.7
C4—C5—H5	118.6	C18—C17—C16	120.4 (2)
C5—C6—C7	116.5 (2)	C18—C17—H17	119.8
C5—C6—H6	121.8	C16—C17—H17	119.8
C7—C6—H6	121.8	C17—C18—C19	120.0 (2)
O—C7—C2	110.71 (18)	C17—C18—H18	120.0
O—C7—C6	126.35 (19)	C19—C18—H18	120.0
C2—C7—C6	122.9 (2)	C18—C19—C20	120.0 (2)
C1—C8—O	110.98 (18)	C18—C19—H19	120.0
C1—C8—C9	134.7 (2)	C20—C19—H19	120.0
O—C8—C9	114.35 (18)	C19—C20—C15	120.9 (2)
C10—C9—C14	118.9 (2)	C19—C20—H20	119.5
C10—C9—C8	120.0 (2)	C15—C20—H20	119.5
C14—C9—C8	121.05 (19)	S—C21—H21A	109.5
C11—C10—C9	121.3 (2)	S—C21—H21B	109.5
C11—C10—H10	119.3	H21A—C21—H21B	109.5
C9—C10—H10	119.3	S—C21—H21C	109.5
C10—C11—C12	118.0 (2)	H21A—C21—H21C	109.5
C10—C11—H11	121.0	H21B—C21—H21C	109.5
			
C21—S—C1—C8	−113.4 (2)	C1—C8—C9—C10	155.3 (3)
C21—S—C1—C2	68.3 (2)	O—C8—C9—C10	−23.3 (3)
C8—C1—C2—C7	−0.2 (3)	C1—C8—C9—C14	−24.7 (4)
S—C1—C2—C7	178.46 (18)	O—C8—C9—C14	156.6 (2)
C8—C1—C2—C3	178.5 (3)	C14—C9—C10—C11	0.8 (4)
S—C1—C2—C3	−2.9 (4)	C8—C9—C10—C11	−179.3 (2)
C7—C2—C3—C4	0.4 (3)	C9—C10—C11—C12	−0.9 (4)
C1—C2—C3—C4	−178.1 (3)	C10—C11—C12—F	−179.8 (2)
C2—C3—C4—C5	−1.6 (3)	C10—C11—C12—C13	0.2 (4)
C2—C3—C4—C15	177.4 (2)	F—C12—C13—C14	−179.3 (2)
C3—C4—C5—C6	1.3 (4)	C11—C12—C13—C14	0.8 (4)
C15—C4—C5—C6	−177.7 (2)	C12—C13—C14—C9	−0.9 (4)
C4—C5—C6—C7	0.2 (4)	C10—C9—C14—C13	0.2 (4)
C8—O—C7—C2	0.2 (3)	C8—C9—C14—C13	−179.8 (2)
C8—O—C7—C6	−179.9 (2)	C3—C4—C15—C20	−150.9 (2)
C3—C2—C7—O	−178.9 (2)	C5—C4—C15—C20	28.0 (4)
C1—C2—C7—O	0.0 (3)	C3—C4—C15—C16	27.4 (3)
C3—C2—C7—C6	1.2 (4)	C5—C4—C15—C16	−153.6 (2)
C1—C2—C7—C6	−180.0 (2)	C20—C15—C16—C17	1.4 (4)
C5—C6—C7—O	178.6 (2)	C4—C15—C16—C17	−177.0 (2)
C5—C6—C7—C2	−1.5 (4)	C15—C16—C17—C18	−0.2 (4)
C2—C1—C8—O	0.3 (3)	C16—C17—C18—C19	−1.6 (4)
S—C1—C8—O	−178.31 (17)	C17—C18—C19—C20	2.3 (4)
C2—C1—C8—C9	−178.4 (3)	C18—C19—C20—C15	−1.2 (4)
S—C1—C8—C9	3.0 (4)	C16—C15—C20—C19	−0.7 (4)
C7—O—C8—C1	−0.3 (3)	C4—C15—C20—C19	177.7 (2)
C7—O—C8—C9	178.73 (19)		
